# Bioactivity‐guided isolation of anti‐inflammatory components from *Phyllanthus emblica*

**DOI:** 10.1002/fsn3.1553

**Published:** 2020-04-22

**Authors:** Wei Li, Hua‐wei Zhu, Yun‐jiao Chen, Hang Xiao, Ya‐zhong Ge, Hai‐e Hu, Xue‐li Li, Yong Cao

**Affiliations:** ^1^ Guangdong Provincial Key Laboratory of Nutraceuticals and Functional Foods Guangdong Research Center for Engineering Technology in Bioactive Natural Products College of Food Science South China Agricultural University Guangzhou China; ^2^ Infinitus (China) Co., Ltd. Guangzhou China; ^3^ Department of Food Science University of Massachusetts Amherst MA USA; ^4^ Eastroc Beverage Group Co., Ltd. Guangdong China

**Keywords:** anti‐inflammation, fisetin, *Phyllanthus emblica*, RAW264.7 macrophages

## Abstract

*Phyllanthus emblica* (*P. emblica*) is a traditionally edible fruit that is good for treatment of biliary diseases, bronchitis, etc. It has obvious anti‐inflammatory activity, but few studies focus on its anti‐inflammatory active substance basis. The purpose of this study was to explore the material basis of anti‐inflammatory activity of *P.* *emblica*, purify, and identify anti‐inflammatory active monomers. Fisetin and gallic acid, which were identified after separation from ethanol extract components of *P. emblica*, exhibited the best anti‐inflammatory effects, markedly inhibiting nitric oxide and proinflammatory cytokine levels in LPS‐stimulated macrophages. In particular, fisetin with significant anti‐inflammatory activity was firstly identified from *P. emblica*. For the first time, our research systematically revealed the material basis of the anti‐inflammatory effects of *P. emblica* from the perspective of the composition of the bioactive substances and provided scientific research methods and ideas for researching bioactive monomers in other plant extracts.

## Graphical abstract

Anti‐inflammatory active monomers from *Phyllanthus emblica* were purified and identified. Fisetin was firstly identified from *Phyllanthus emblica*. Fisetin and gallic acid seem to be at least particially responsible for anti‐inflammatory effect of *Phyllanthus emblica*.

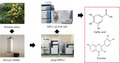

## 1 INTRODUCTION

Inflammation is a series of complex physiological changes that the body undergoes in response to external stimuli. With the emergence of drug resistance and adverse reactions to synthetic anti‐inflammatory drugs, development has been initiated on Chinese herbal medicines with abundant resources, few side effects, and good curative effects. The development of natural anti‐inflammatory drugs has become a research hot spot. *Phyllanthus emblica* is a traditionally edible fruit belonging to the genus Euphorbia that has been widely used in traditional medicine systems, such as traditional Chinese, Tibetan, and Indian medicine (Liu et al., 2008; Zhang, Tanaka, Iwamoto, Yang, & Kouno, 2000). As a medicine and food plant, *P. emblica* is rich in vitamins, trace elements and minerals, amino acids, and superoxide dismutase (Barthakur & Arnold, 1991; Nisar et al., 2018). In recent years, there have been many reports concerning the biological activities of *P. emblica*, including its anti‐inflammatory (Dang, Parekar, Kamat, Scindia, & Rege, 2011; Kiemer, Hartung, Huber, & Vollmar, 2003; Sripanidkulchai & Junlatat, 2014), antitumor (Huang et al., 2006; Zhao, Sun, Marques, & Witcher, 2015), and antioxidant (Liu et al., 2008; Poltanov et al., 2009; Rose, Wan, Thomas, Seeram, & Ma, 2018) activities. Most studies have focused on the biological activities of the *P. emblica* ethanol extract (PEE), but few studies have characterized the bioactive constituents responsible for its pharmacological effects. Our previous research screened PEE for the most anti‐inflammatory active components based on significant inhibition of secretion of nitric oxide (NO) and cytokines by RAW264.7 macrophages. The aim of the present study was to investigate the material basis of this anti‐inflammatory effect using PEE as the target for this research. Experiments were carried out to isolate and purify PEE using a normal‐phase silica gel column and prep‐high‐performance liquid chromatography (HPLC). Lipopolysaccharide (LPS)‐stimulated RAW264.7 macrophages were used as a model of inflammation with which to identify the most anti‐inflammatory components based on the amounts of NO and cytokines (TNF‐α, IL‐1β, and IL‐6) released as indicators. Meanwhile, HPLC/quadrupole time‐of‐flight mass spectrometry (Q‐TOF/MS) technology was used to preliminarily identify active anti‐inflammatory substances. This research evaluated the most anti‐inflammatory components of PEE and determined the primary composition of the extracts.

## 2 MATERIALS AND METHODS

### 2.1 Reagents and materials

RAW264.7 mouse macrophages were purchased from the Kunming Cell Bank of the Chinese Academy of Sciences Culture Collection. The dried fruits of *Phyllanthus emblica* were provided by Infinitus (China) Co., Ltd. LPS and dexamethasone were purchased from Sigma‐Aldrich Co., Dulbecco's Modified Eagle's Medium (DMEM) was purchased from Hyclone, fetal bovine serum was purchased from Gibco BRL, NO test kit was obtained from Beyotime Biotechnology and the enzyme immunoassay kits for IL‐1, IL‐6, and TNF‐α were purchased from Neobioscience Technology Co., Ltd. All other reagents were of analytical grade.

### 2.2 Preparation of extract from *P. emblica* and bioactivity‐guided isolation

Dried fruits of *P. emblica* (501.32 g) were subjected to extraction twice with 70% EtOH by boiling for 2 hr (4 L) and 1.5 hr (3 L), respectively, and the extracts were combined. After filtration, the extracts were evaporated under vacuum and dried using vacuum freeze dryer. The 214.32 g extract () was obtained. Part of the extract (120.05 g) was dissolved in 70% EtOH and mixed with normal‐phase silica gel (240 g), then subjected to silica gel (C18, 15 μm) glass column (16 cm × 120 cm), and eluted using a range of different polar eluent systems, including petroleum ether (PE, 40 L), PE: ethyl acetate (EAC) 5:5 (40 L), PE: EAC 1:9 (40 L), EAC: methyl alcohol (MT) 9:1 (40 L), EAC:MT 5:5 (40 L), EAC:MT 1:9 (40 L), and EAC:MT:H2O 1:5:4 (40 L). Seven eluent systems were used for elution, where three column volumes of 500 ml were used for each system. Based on TLC, twelve elution fractions were obtained and designated a‐l.

The fractions c (1.01 g) with the best anti‐inflammatory activity was separated by prep‐HPLC (LC‐8A, Shimadzu). A flow rate of 20 ml/min was used to elute samples with a gradient mode of A (0.1% formic acid: water) and B (methanol): 0–6 min 5%–7%, 6–16 min 7%–30%, 16–31 min 30%–80%, and 31–40 min 80%–90%. The injection volume was 2 ml, and the column temperature was set to 40°C. Two fractions c1 (420 mg) and c2 (545 mg) were obtained. Fraction c1 was determined to be a mixture of gallic acid and fisetin and demonstrated to be of a stronger anti‐inflammatory activity than fraction c2 using an LPS‐stimulated RAW 264.7 macrophage anti‐inflammation model.

### 2.3 Cell culture

RAW264.7 mouse macrophages were cultured in DMEM complete medium containing 10% fetal bovine serum, 100 U/ml penicillin, and 100 μg/ml streptomycin at 37°C in a humidified atmosphere of 5% CO2. The culture medium was replaced every 1–2 days, and the cells were passaged every 4–6 days (Jung, Jin, Ahn, Lee, & Choi, 2013).

### 2.4 Measurement of NO, IL‐1, IL‐6, and TNF‐α production

RAW264.7 cell suspension was added to a 96‐well plate (5.0 × 103 cells/100 µl/well), incubated at 37°C in a 5% CO2 incubator for 4 hr, and then stimulated with LPS (1 μg/ml) for 24 hr. After another 24‐hr treatment with different concentrations of compounds, the NO levels were determined by measuring the nitrite levels in the supernatant using the Griess reagent assay (Tursun et al., 2016), and the IL‐1, IL‐6, and TNF‐α levels in the supernatant were determined using enzyme immunoassay kits.

### 2.5 HPLC‐Q/TOF‐MS conditions

HPLC‐mass spectrometry (MS) technology was used for analysis, where HPLC was used to separate the chemical components of the sample and MS was used to identify the structures. An Agilent 6550 iFunnel Q‐TOF LC/MS system was used to analyze the samples. A flow rate of 1 ml/min was used to elute samples with a gradient mode of A (0.1% formic acid: water) and B (methanol): 0–6 min 5%–7%, 6–16 min 7%–30%, 16–31 min 30%–80%, and 31–40 min 80%–90%. The injection volume was 5 μl, and the column temperature was set to 40°C.

MS conditions: ion source was dual‐spray, multi‐reaction detection was in negative ion mode, and the scanning range was 105–1100 *m/z*. The gas path parameters included the use of dry nitrogen, a flow rate of 9 L/min, a drying gas temperature of 350°C, an air curtain of 50 Psi, a nozzle voltage of 500 V, and a capillary outlet voltage of 150 V.

### 2.6 Statistical analysis

All the data were analyzed by ANOVA (SPSS, 2010) and are expressed as mean ± standard deviations. The differences among treatments were examined by LSD and Duncan's multiple‐range tests, and *p* < .05 and *p* < .01 were considered statistically significant.

## 3 RESULTS

### 3.1 Effect of PEE elution fractions on production of NO and cytokines (TNF‐a, IL‐6, and IL‐1β) in LPS‐stimulated RAW 264.7 macrophages

#### 3.1.1 Effect of PEE elution fractions on production of NO in LPS‐stimulated RAW 264.7 macrophages

NO is an important bioinformatics molecule and has dual roles as a bio‐messenger and cytotoxic molecule. It plays a part in the pathogenicity of inflammation and is an indicator of inflammation due to being overexpressed in macrophages stimulated by LPS, TNF‐γ, and other substances (Hortelano, Zeini, & Boscá, 2002; Libby et al., 2018). As shown in Table 1, the NO production from RAW264.7 macrophages in the LPS‐stimulated model group was significantly higher than that in the control group (*p* < .01). This showed the macrophages exhibited an inflammatory response to LPS stimulation and an anti‐inflammatory model was successfully established. When the sample dose was 50 μg/ml, the NO levels in groups f, i, j, and k were significantly lower than the model group by 57.02, 77.63, 67.90, and 54.57%, respectively (*p* < .01). Compared with the model group, groups i and j at the 6.25 μg/ml dose displayed weak anti‐inflammatory activity as groups d and j did at a dose of 400 μg/ml (*p* > .05). Meanwhile, other groups promoted inflammation. In addition, as presented in Table 1, NO was easily detectable in most groups at dose of 6.25 μg/ml, while 400 μg/ml dose groups had higher NO levels than the model group. This revealed the PEE elution fractions have optimal doses to exert anti‐inflammation activity. Concentrations of samples that were excessive or too low could affect the anti‐inflammatory activity because toxicity itself might be able to stimulate proinflammatory mediator production instead of anti‐inflammation activity. In particular, the four groups f, i, j, and k could be selected as alternative components for later research.

**TABLE 1:** Effects of different cross‐column components from PEE on NO release by LPS‐stimulated RAW264.7 cells (x ± s, *n* = 3)

Dose group (μg/ml)	6.25	50	400
Control	0.71 ± 0.19	0.71 ± 0.19	0.71 ± 0.19
Model group	9.15 ± 2.12^$$^	9.15 ± 2.12^$$^	9.15 ± 2.12^$$^
Positive group	4.60 ± 1.00*	4.60 ± 1.00*	4.60 ± 1.00*
a	12.04 ± 2.01	7.60 ± 0.67	19.38 ± 1.71**
b	10.60 ± 2.18	13.38 ± 0.96*	44.05 ± 9.05**
c	12.82 ± 1.84*	8.15 ± 1.68	34.38 ± 10.14**
d	13.38 ± 1.02*	6.15 ± 2.91	7.60 ± 0.67
e	11.16 ± 0.51	7.82 ± 2.36	22.60 ± 3.79**
f	11.27 ± 2.00	3.93 ± 0.34**	11.93 ± 0.67
g	12.16 ± 0.20	8.82 ± 4.76	10.82 ± 0.39
h	14.38 ± 0.51**	5.82 ± 2.14	11.05 ± 1.35
i	8.60 ± 0.88	2.05 ± 2.27**	9.27 ± 1.77
j	9.04 ± 2.22	2.94 ± 1.16**	7.27 ± 1.73
k	12.04 ± 4.82	4.16 ± 0.51**	12.49 ± 2.59
l	14.38 ± 3.37**	6.38 ± 3.10	9.27 ± 0.34

Abbreviations: LPS, lipopolysaccharide; NO, nitric oxide; PEE, Phyllanthus emblica ethanol extract.

Model group versus control group:

Sample group versus Model group:

^$^ *p* < .05,

^$$^ *p* < .01;

* *p* < .05,

** *p* < .01.

#### 3.1.2 Effect of PEE elution fractions on TNF‐a, IL‐6, and IL‐1β production in LPS‐stimulated RAW264.7 macrophages

Proinflammatory cytokines, such as TNF‐a, IL‐1β, and IL‐6, were identified as markers of anti‐inflammation and could be released by LPS‐stimulated macrophages (Lee et al., 2011; Park et al., 2012; Tursun et al., 2016). As presented in Table 2, the TNF‐α levels in groups a, b, c, d, e, f, g, and h were significantly reduced by 29.95, 46.77, 43.14, 28.99, 39.49, 43.67, 33.14, and 22.25%, respectively, compared with the model group when the sample concentration was 6.25 μg/ml (*p* < .01). In addition, a 50 μg/ml dose of each group, with the exception of h, resulted in significant anti‐inflammatory activity (*p* < .01). The group a had the greatest inhibition rate of 54.49%, followed by group b with an inhibition rate of 41.86%. The TNF‐α levels of groups b, c, i, k, and l at sample concentration of 400 μg/ml were 41.50, 35.38, 43.38, 24.84, and 39.20% lower than the model group (*p* < .05).

**TABLE 2:** Effects of different cross‐column components from PEE on TNF‐α release by LPS‐stimulated RAW264.7 cells (x ± s, *n* = 3)

Dose group (μg/ml)	6.25	50	400
Control	190.57 ± 47.03	190.57 ± 47.03	190.57 ± 47.03
Model group	2,869.62 ± 114.43^$$^	2,869.62 ± 114.43^$$^	2,869.62 ± 114.43^$$^
Positive group	847.24 ± 52.88**, $	847.24 ± 52.88**, $	847.24 ± 52.88**, $
a	2010.10 ± 217.17**, $	1,305.81 ± 235.18**, $	3,532.48 ± 432.74**, $
b	1631.52 ± 198.10**, $	1668.19 ± 159.02**, $	1854.38 ± 15.14*
c	1527.24 ± 86.77**, $	1733.90 ± 107.29**, $	1678.67 ± 112.13**, $
d	2037.71 ± 40.73**, $	1,860.57 ± 347.65**, $	4,052.00 ± 337.68**, $
e	1736.29 ± 68.76**, $	1729.14 ± 216.60**, $	4,544.38 ± 115.36**, $
f	1616.29 ± 235.59**, $	1775.81 ± 265.48**, $	3,753.90 ± 160.82**, $
g	1918.67 ± 31.83**, $	1965.81 ± 120.50**, $	2,560.57 ± 78.88**, $
h	2,231.05 ± 159.73**, $	2,536.29 ± 472.21	3,343.43 ± 526.54**, $
i	3,106.76 ± 538.76	1,850.57 ± 381.97**, $	1624.86 ± 327.88**, $
j	2,679.62 ± 513.04	2,181.52 ± 239.78**, $	3,070.57 ± 283.12**, $
k	2,859.62 ± 324.49	2017.24 ± 129.09**, $	2,156.76 ± 250.32**, $
l	2,826.76 ± 63.73	2,293.43 ± 177.16**, $	1744.86 ± 146.61**, $

Note

Model group versus control group:

Sample group versus Model group:

^$^ *p* < .05,

^$$^ *p* < .01;

* *p* < .05,

** *p* < .01.

TNF‐α, IL‐6, and IL‐1β cytokines are important indicators of cellular inflammatory response, of which TNF‐α causes severe inflammatory injury to cells and can induce synergistic effects with IL‐1β and IL‐6. These proinflammatory cytokines accelerate the arachidonic acid metabolism and cause excessive inflammation (Harun, Vidyadaran, Lim, Cole, & Ramasamy, 2015; Wang et al., 2011). In our study, as shown in Table 3, all PEE groups caused a decrease in IL‐1β release by LPS‐stimulated macrophages compared with the model group, demonstrating remarkable anti‐inflammatory activity (*p* < .01). Based on the inhibitory effect at the three concentrations described above, groups c, g, and h were initially selected as the best anti‐inflammatory candidates based on the IL‐1β levels. Likewise, IL‐6 is a vital proinflammatory cytokine that regulates inflammatory responses (Lee et al., 2011; Tung, Huang, Lin, & Yen, 2018). Table 4 shows that the IL‐6 levels were dramatically suppressed in LPS‐stimulated macrophages treated with the PEE groups (*p* < .01). In particular, groups e, f, and g had the best anti‐inflammatory activity at 6.25 and 50 μg/ml, while groups b and c significantly inhibited IL‐6 secretion by 83.34 and 82.86% at 400 μg/ml, respectively.

**TABLE 3:** Effects of different cross‐column components from PEE on IL‐1β release by LPS‐stimulated RAW264.7 cells (x ± s, *n* = 3)

Dose group (μg/ml)	6.25	50	400
Control	35.28 ± 2.02	35.28 ± 2.02	35.28 ± 2.02
Model group	78.78 ± 2.02^**^, ^$^	78.78 ± 2.02^**^, ^$^	78.78 ± 2.02^**^, ^$^
Positive group	48.28 ± 2.75**	48.28 ± 2.75**	48.28 ± 2.75**
a	56.40 ± 0.78**	52.78 ± 10.80**	43.45 ± 6.87**
b	48.62 ± 5.48**	47.78 ± 9.36**	35.45 ± 3.46**
c	35.95 ± 6.56**	41.95 ± 4.27**	32.95 ± 4.09**
d	42.28 ± 6.25**	43.45 ± 3.50**	34.62 ± 6.29**
e	48.62 ± 4.62**	49.45 ± 9.73**	43.45 ± 2.00**
f	41.62 ± 1.53**	43.95 ± 3.12**	37.12 ± 2.57**
g	43.45 ± 5.29**	36.12 ± 2.25**	34.45 ± 1.32**
h	41.95 ± 1.80**	36.62 ± 0.29**	34.62 ± 3.40**
i	46.95 ± 7.55**	43.95 ± 2.00**	48.28 ± 7.82**
j	45.78 ± 4.25**	49.62 ± 5.03**	47.62 ± 3.01**
k	40.28 ± 3.75**	41.12 ± 3.01**	38.95 ± 2.65**
l	44.12 ± 5.01**	36.28 ± 5.51**	42.28 ± 6.93**

Note

Model group versus control group:

Sample group versus Model group:

^$^ *p* < .05,

^$$^ *p* < .01;

* *p* < .05,

** *p* < .01.

**TABLE 4:** Effects of different cross‐column components from PEE on IL‐6 release by LPS‐stimulated RAW264.7 cells (x ± s, *n* = 3)

Dose Group (μg/ml)	6.25	50	400
Control	164.08 ± 20.53	164.08 ± 20.53	164.08 ± 20.53
Model group	864.68 ± 21.54^$$^	864.68 ± 21.54^$$^	864.68 ± 21.54^$$^
Positive group	304.38 ± 13.77**	304.38 ± 13.77**	304.38 ± 13.77**
a	615.89 ± 50.15**	542.11 ± 61.94**	299.83 ± 19.24**
b	586.20 ± 0.95**	510.59 ± 6.15**	144.08 ± 2.29**
c	614.83 ± 64.69**	478.47 ± 30.76**	148.17 ± 3.92**
d	729.98 ± 128.95**	545.44 ± 84.47**	266.20 ± 9.58**
e	475.59 ± 80.05**	381.20 ± 69.48**	232.86 ± 47.75**
f	460.44 ± 43.12**	414.08 ± 32.21**	282.26 ± 11.59**
g	498.62 ± 18.92**	510.59 ± 80.87**	274.38 ± 3.87**
h	569.68 ± 110.28**	534.53 ± 75.60**	387.11 ± 21.07**
i	717.56 ± 57.85**	662.11 ± 12.47**	441.95 ± 50.62**
j	608.62 ± 22.37**	640.59 ± 10.15**	409.83 ± 8.93**
k	748.62 ± 49.51*	751.95 ± 65.10*	478.32 ± 24.91**
l	776.65 ± 31.20	849.98 ± 71.64a	672.11 ± 23.03**

Note

Model group versus control group:

Sample group versus Model group:

^$^ *p* < .05,

^$$^ *p* < .01;

* *p* < .05,

** *p* < .01.

NO and TNF‐α are two major indicators of inflammatory injury; TNF‐α causes dramatic inflammatory damage that could induce and synergize with the activity of other cytokines, such as IL‐1β and IL‐6. The present study showed that components f, i, j, and k at a dose of 50 μg/ml exhibited the strongest anti‐inflammatory activity based on NO secretions by RAW 264.7 macrophages. Based on TNF‐α secretions, components b and c had the best anti‐inflammatory activity at different concentrations. Meanwhile, components b and c also had significant anti‐inflammatory activity based on the IL‐1β and IL‐6 indicators. Based on the above results in terms of both inhibitory effect and effective concentrations, components b and c were selected for further comparison. As shown in Tables 2 and 3, the component c at a dose of 400 μg/ml for the TNF‐α indicator and 6.25 μg/ml for the IL‐1β indicator both exhibited more significant anti‐inflammatory activity than the component b (*p* < .05). Therefore, the component c was selected for the subsequent experiments.

### 3.2 Effect of c1 and c2 on production of NO and cytokines (TNF‐a, IL‐6, and IL‐1β) in LPS‐stimulated RAW 264.7 macrophages

#### 3.2.1 Effect of c1 and c2 of c elution fraction on NO production by LPS‐stimulated RAW 264.7 macrophages

**FIGURE 1:**
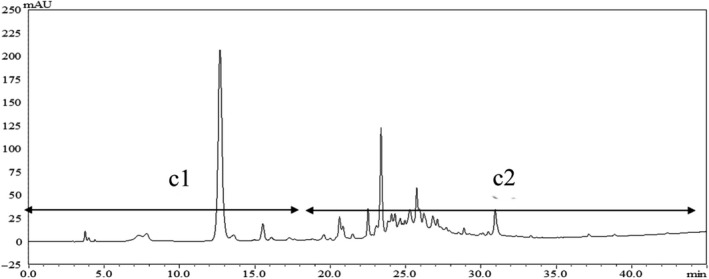
High‐performance liquid chromatogram of c component (c1 0–18 min, c2 18–45 min)

**FIGURE 2:**
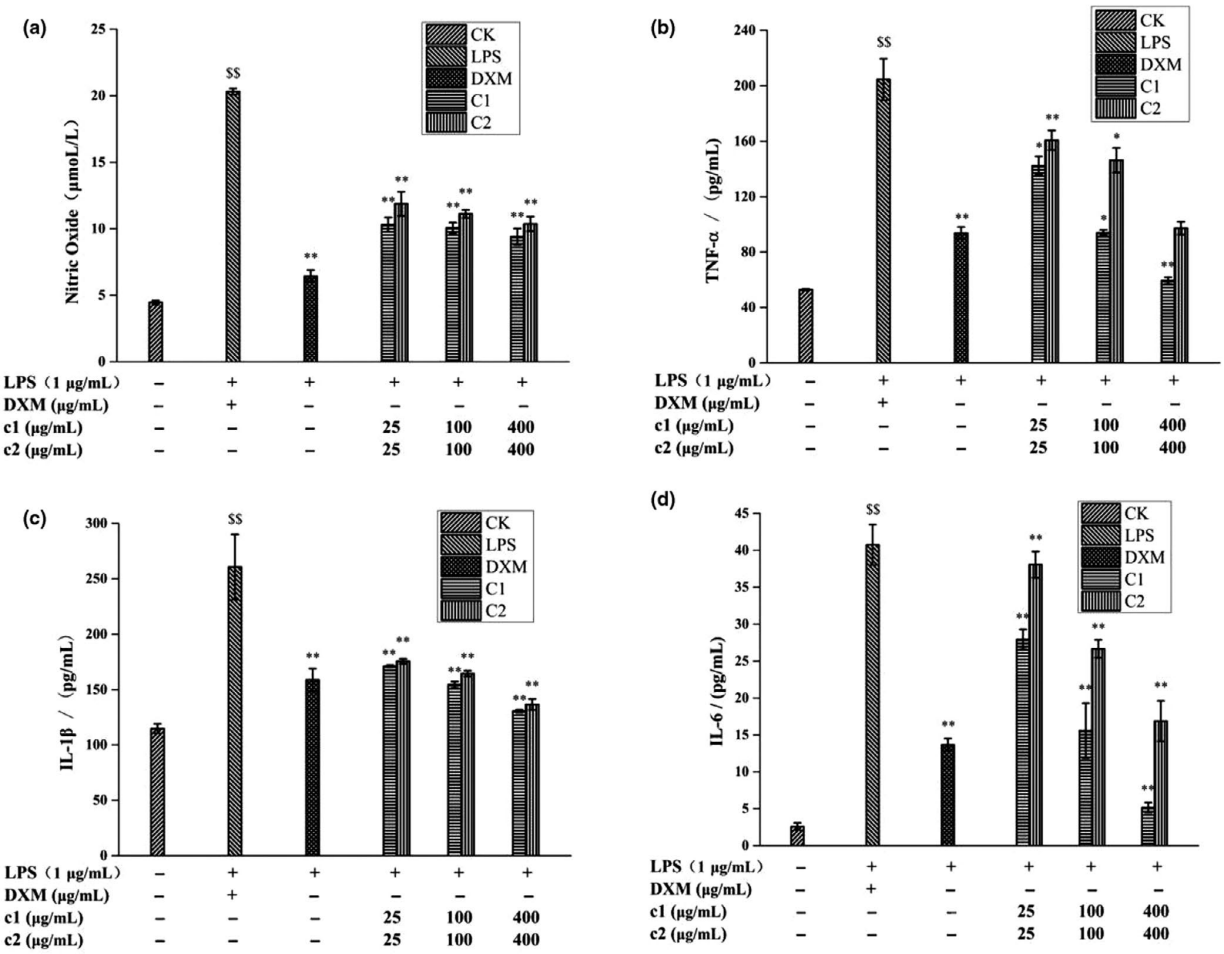
Anti‐inflammatory effects of c1 and c2 by LPS‐stimulated RAW264.7 cells ((a) Effects of c1 and c2 on NO release by LPS‐stimulated RAW264.7 cells; (b) Effects of c1 and c2 on TNF‐α release by LPS‐stimulated RAW264.7 cells; (c) Effects of c1 and c2 on IL‐β release by LPS‐stimulated RAW264.7 cells; (d) Effects of c1 and c2 on IL‐6 release by LPS‐stimulated RAW264.7 cells; Model group versus control group: $ *p* < .05, $$ *p* < .01; Sample group versus Model group: * *p* < .05, ** *p* < .01)

The component c was separated and purified by prep‐HPLC, concentrated in a vacuum, and then lyophilized to obtain samples c1 and c2 (Figure 1), which were then evaluated in a macrophage inflammation model. As shown in Figure 2a, c1 and c2 were notably anti‐inflammatory in a concentration‐dependent manner. The anti‐inflammatory activity of c1 was higher than that of c2 with the most inhibition of NO levels of 53.72% by c1 at 400 μg/ml.

### 3.3 Effect of c1 and c2 of c elution fractions on TNF‐a, IL‐6, and IL‐1β production by LPS‐stimulated RAW264.7 macrophages

Statistically significant changes in TNF‐α, IL‐1β, and IL‐6 levels were observed (*p* < .01) when comparing the model group with the control group, indicating the success of the model. Figure 2b illustrates that c1 and c2 significantly inhibited the release of TNF‐α from LPS‐stimulated RAW264.7 macrophages compared with the model group, and c1 was a better inhibitor than c2 at different concentrations (*p* < .05). Furthermore, c1 exhibited the best anti‐inflammatory activity at 400 μg/ml, causing a 70.95% decrease, which was comparable to that induced by 100 μg/ml dexamethasone. Similarly, Figure 2c,d show that c1 and c2 suppressed the production of IL‐1β and IL‐6 in LPS‐stimulated RAW264.7 cells in a concentration‐dependent manner and the anti‐inflammatory effect of c1 was also stronger than that of c2. These results indicate c1 should be a target of subsequent material identification.

### 3.4 Characterization, validation, and anti‐inflammatory activity of c1

**FIGURE 3:**
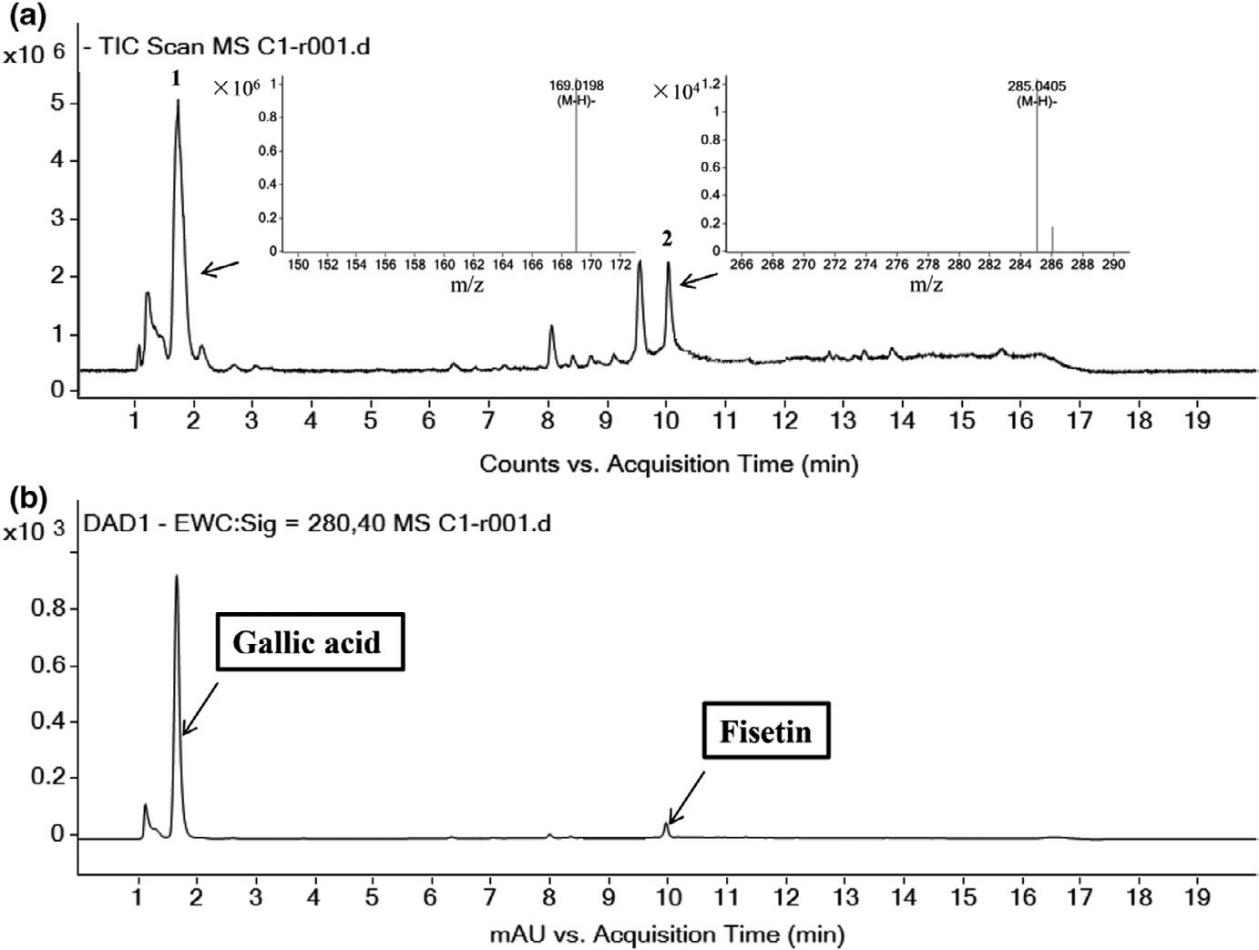
Total ion chromatogram for c1 obtained under negative mode

**FIGURE 4:**
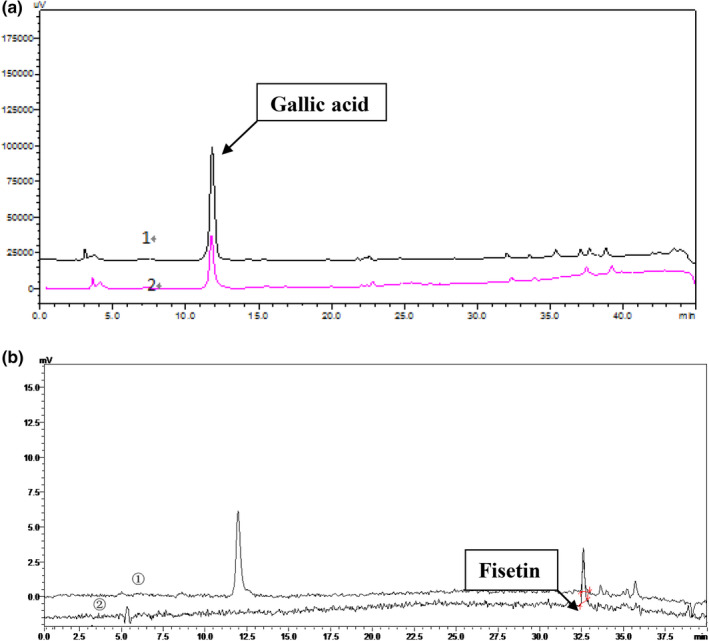
The spectrometry results of c1 component verified by HPLC ((a) High‐performance liquid chromatograms of c1 and gallic acid standard, 1 c1 component, 2 gallic acid standard; (b) High‐performance liquid Chromatograms of c1 and fisetin standard, ① c1 component, ② fisetin standard)

An HPLC‐Q‐TOF‐MS method was established to characterize the chemical components of c1. A typical total ion chromatogram and HPLC chromatogram are presented in Figure 3. As shown in Figure 3a, the mass spectra of compounds 1 and 2 in negative mode HPLC‐Q/TOF‐MS showed the (M‐H) ‐ ion at *m/z* 169.0159 and 285.0405, respectively; therefore, these two compounds were initially identified as gallic acid and fisetin (Luo, Zhao, Yang, Shen, & Rao, 2009), and their relevant information is shown in Table 5. The gallic acid and fisetin standards were then used to qualitatively detect c1 components by HPLC to further verify the spectrometry results (Yang, Kortesniemi, Liu, Karonen, & Salminen, 2012). As shown in Figure 4 a,b, the component c1 was initially determined to contain gallic acid and fisetin by comparing the retention time with that of the corresponding standards under the same conditions, respectively.

**FIGURE 5:**
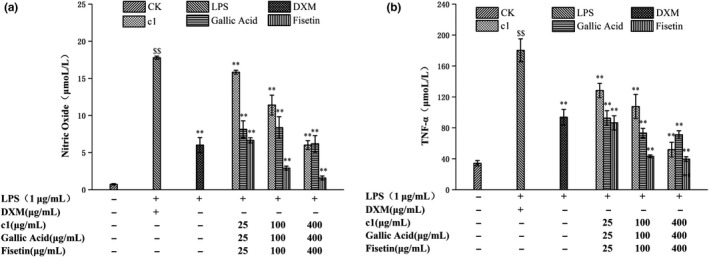
Anti‐inflammatory effects of c1, gallic acid, and fisetin evaluated by LPS‐stimulated RAW264.7 cells ((a) Effects of c1, gallic acid, and fisetin on NO release by LPS‐stimulated RAW264.7 cells; (b) Effects of c1, gallic acid, and fisetin on TNF‐α release by LPS‐stimulated RAW264.7 cells; Model group versus control group: $ *p* < .05, $$ *p* < .01; Sample group versus Model group: * *p* < .05, ** *p* < .01)

To further study the contribution of anti‐inflammatory active monomers to the anti‐inflammatory effects of c1 components, c1, gallic acid and fisetin purified from c1 were evaluated for anti‐inflammatory activity based on NO and TNF‐α production. As shown in Figure 5a, with an increase in concentration, the effect of fisetin on NO secretion by macrophages increased gradually with inhibition rates of 62.68, 83.68, and 91.11% at concentrations of 25 µg/ml, 100 µg/ml, and 400 µg/ml, respectively (*p* < .01). Additionally, gallic acid and component c1 significantly inhibited the secretion of NO by macrophages in a concentration‐dependent manner (*p* < .05), and gallic acid exhibited better anti‐inflammatory effects at 25 µg/ml and 100 µg/ml. However, the inhibition rate of the component c1 at 400 µg/ml (66.24%) was slightly higher than that of the gallic acid (63.29%) at the same concentration. Moreover, fisetin at 400 µg/ml also exhibited a significant inhibition on NO production. These results suggest that both gallic acid and fisetin contribute to the anti‐inflammatory activity of c1. Similar results were also observed for TNF‐α production. It can be seen from Figure 5b that c1, gallic acid, and fisetin all exhibited obvious inhibitory effect on TNF‐α secretion by macrophages when compared with the LPS group (*p* < .05) with inhibition rates of 60.52, 78.00, and 71.26% for 400 µg/ml of gallic acid, fisetin, and c1, respectively. Taken together, these results suggest that the anti‐inflammatory activity of the component c1 at least results from gallic acid and fisetin.

## 4 DISCUSSION

Macrophages are involved in the majority of inflammatory reactions involving stimulation with LPS or other stimuli and can secrete proinflammatory cytokines, such as NO, TNF‐α, IL‐1β, and IL‐6 (Linton & Fazio, 2003; Wang et al., 2017). Modern studies indicate natural products may inhibit the inflammatory response by regulating NO or inflammatory factors in macrophages (Fang, Rao, & Tzeng, 2008). Of these natural products, saponins and polyphenols were found to be the main anti‐inflammatory active ingredients in an in‐depth study of homologous medicine and food plants (Zhang et al., 2011), which acted mainly by inhibiting inflammatory mediators, the NF‐κβ signaling pathway, and COX‐2 expression (Kumar, 2014; Zhang et al., 2017). *P. emblica* leaf, branch, and fruit extracts were verified to be of anti‐inflammatory activity by inhibiting proinflammatory cytokines NO, TNF‐α, IL‐6, etc (Perianayagam, Sharma, Joseph, & Christina, 2004; Wang et al., 2017). However, few reports focused on identification of anti‐inflammatory components in *P. emblica* extracts. In the present study, normal‐phase silica gel column chromatography and preparative liquid chromatography were combined to isolate anti‐inflammatory compounds from *P. emblica*. Similar research purified the ethyl acetate fraction of emblica fruit extract using silica gel columns and thin‐layer chromatography and identified six compounds by MS and nuclear magnetic resonance spectroscopy, including gallic acid, quercetin, and cinnamic acid (Luo et al., 2009). In this study, gallic acid and fisetin derived from fruits of *P. emblica* were identified using HPLC‐Q/TOF‐MS. Similar results were obtained by Luo et al. (Luo et al., 2011). Notably, herein, for the first time, we reported the existing of fisetin in *P. emblica*. Further research revealed that gallic acid and fisetin both exhibited remarkable anti‐inflammatory activity in LPS‐induced RAW 264.7 cells model, which is in agreement with other reports (Fei, Je, Shin, Kim, & Seo, 2017; Feng, Jiang, Sun, Fu, & Li, 2017; Seo, Jeong, Yoo, Lee, & Shin, 2016). Additionally, gallic acid from the ethyl acetate fraction of Punica granatum could suppress LPS‐induced release of NO, PGE‐2, and IL‐6, thereby contributing to anti‐inflammatory effects of the extracts (Wang et al., 2017). Fisetin also exhibited effective anti‐inflammatory capacity in relieving LPS‐induced acute lung injury in rats by regulating TLR4‐mediated NF‐kappaB signaling pathway (Feng et al., 2017).

Our results demonstrated that fractions of *P. emblica* fruit EtOH extracts exhibited different degree of anti‐inflammatory capacity in suppressing NO, TNF‐α, IL‐1β, and IL‐6 production. The two isolated anti‐inflammatory compounds, gallic acid and fisetin, exhibited similar inhibitory effects. Therefore, we presumed gallic acid and fisetin might be responsible for the anti‐inflammatory effect of *P. emblica* (BenSaad L. A et al., 2017). It is worth noting that gallic acid content (over 1.2%) is considered as quality control of *P. emblica* according to the Chinese pharmacopoeia (2015). In the present study, the gallic acid content in *P. emblica* extracts was found to be significantly higher than that of fisetin, while the anti‐inflammatory effect of the fractions of *P. emblica* extracts might be attributed to both gallic acid and fisetin. Further studies into the anti‐inflammatory mechanism of fisetin and the isolation of other anti‐inflammatory compounds in *P. emblica* could be conducted in the future.

## 5 CONCLUSIONS

In summary, this study showed that silica gel columns, thin‐layer chromatography, HPLC, and HPLC‐Q‐TOF‐MS could be effectively combined to analyze and identify anti‐inflammatory active substances in PEE using a macrophage model for evaluating anti‐inflammatory activity. As a result, gallic acid and fisetin were two anti‐inflammatory active monomers derived from PEE that efficiently inhibited production of NO and other inflammatory factors. Of these, this was the first identification of fisetin in PEE and demonstration of its strong anti‐inflammatory activity, which provides a theoretical basis for the use of *P. emblica* in anti‐inflammation.

## CONFLICT OF INTEREST

The authors declared no conflict of interest.

## ETHICAL STATEMENT

This study does not involve any human or animal testing.

6

**TABLE 5:** Compounds identified in c1 by HPLC‐Q/TOF‐MS

RT	Molecular formula	Mass error (ppm)	Tentative assignment	Structural formula	Ref. mass	Obs. mass
1.639	C7H6O5	8.76	Gallic acid	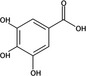	170.0215	170.0271
10.239	C15H10O6	0.26	Fisetin	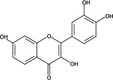	286.0477	286.0478
